# Training to improve contrast sensitivity in amblyopia: correction of high-order aberrations

**DOI:** 10.1038/srep35702

**Published:** 2016-10-18

**Authors:** Meng Liao, Haoxing Zhao, Longqian Liu, Qian Li, Yun Dai, Yudong Zhang, Yifeng Zhou

**Affiliations:** 1 Department of Optometry and Visual Science, West China Hospital, Sichuan University, Chengdu, China; 2 Department of Ophthalmology, West China Hospital, Sichuan University, Chengdu 610041, China; 3 Institute of Optics and Electronics, Chinese Academy of Sciences, Chengdu, China; 4 The Key Laboratory on Adaptive Optics, Chinese Academy of Science, Chengdu 610209, China; 5 CAS Key Laboratory of Brain Function and Disease and School of Life Sciences, University of Science and Technology of China, Hefei 230027, China

## Abstract

Perceptual learning is considered a potential treatment for amblyopia even in adult patients who have progressed beyond the critical period of visual development because adult amblyopes retain sufficient visual plasticity. When perceptual learning is performed with the correction of high-order aberrations (HOAs), a greater degree of neural plasticity is present in normal adults and those with highly aberrated keratoconic eyes. Because amblyopic eyes show more severe HOAs than normal eyes, it is interesting to study the effects of HOA-corrected visual perceptual learning in amblyopia. In the present study, we trained twenty-six older child and adult anisometropic amblyopes while their HOAs were corrected using a real-time closed-loop adaptive optics perceptual learning system (AOPL). We found that adaptive optics (AO) correction improved the modulation transfer functions (MTFs) and contrast sensitivity functions (CSFs) of older children and adults with anisometropic amblyopia. When perceptual learning was performed with AO correction of the ocular HOAs, the improvements in visual function were not only demonstrated in the condition with AO correction but were also maintained in the condition without AO correction. Additionally, the learning effect with AO correction was transferred to the untrained visual acuity and fellow eyes in the condition without AO correction.

Amblyopia is a developmental disorder of spatial vision that results from abnormal visual experience (i.e., anisometropia, strabismus or both) during childhood^1^,^2^. Clinical treatments, including optical correction, patching (i.e., occlusion of the unaffected eye), atropine drops and oral levodopa (see Desantis for a recent review^3^), are effective for child amblyopes but not for older child or adult amblyopes^4^,^5^,^6^. This age-dependence is believed to result from diminishing visual plasticity in people beyond a critical period of visual development^7^.

Recently, several studies have demonstrated that adult amblyopes retain sufficient visual plasticity such that intensive training (i.e., perceptual learning) can be used as a potential treatment for improving their visual performance (see Levi and Li^8^, Astle *et al*.^9^ for reviews). Several training tasks, including vernier acuity^10^,^11^, positional acuity^12^,^13^,^14^, and contrast sensitivity^15^,^16^,^17^ tasks, have been introduced to improve amblyopes’ visual functions. In some cases, training also elicits benefits in untrained tasks, including visual acuity (VA)^15^,^16^,^17^, stereoacuity and visual counting^12^,^14^ tasks. These transfers of learning effects are particularly interesting because they are critical in the clinical practice of treating amblyopia. Recent studies have suggested that a greater degree of neural plasticity is present, and thus greater visual benefits can be achieved in normal adults^18^ and adults with highly aberrated keratoconic eyes^19^,^20^ if perceptual learning is performed in environments free of high-order aberrations (HOAs). These findings indicate that optical quality may play an important role in visual perceptual learning. HOAs refer to optical aberrations such as comas, spherical aberrations, trefoils, etc. that are present in all human eyes and are not correctable with conventional glasses or contact lenses^21^. There is considerable evidence demonstrating that HOAs are relevant to the development of amblyopia^22^,^23^,^24^,^25^,^26^, and amblyopic eyes typically show more severe HOAs^27^.

Given the framework mentioned above, we sought to determine the effects of HOA-corrected visual perceptual learning in amblyopia. To address this issue, we trained twenty-six older child and adult anisometropic amblyopes while their HOAs were corrected using a real-time closed-loop adaptive optics perceptual learning system (AOPL)^28^. A contrast detection task conducted at the individual’s cut-off spatial frequency was used as the training protocol. This task was similar to those in previously reported studies^16^,^17^,^18^,^29^,^30^,^31^ and enabled us to directly compare the learning benefits among different studies. We found that the AOPL system worked well in terms of correcting HOAs, and the improvements in visual function induced by perceptual learning with adaptive optics (AO) correction were maintained in the condition not only with AO correction but also without AO correction. Meanwhile, the untrained visual acuity and visual function of the fellow eyes were also improved. However, compared with previously reported results based on the use of traditional HOA-uncorrected training procedures^16^,^17^,^29^,^31^, this training protocol did not produce more promising visual improvements. The possible reasons for this phenomenon and the clinical significance of HOA-corrected training in amblyopia are discussed.

## Results

All subjects underwent pre- and post-training tests of contrast sensitivity function (CSF) in the amblyopic eyes without and with adaptive optics (AO) correction. The wave-front aberrations were measured during the CSF tests and training sessions and used to calculate the horizontal modulation transfer functions (MTFs)^32^ under the two conditions. After the pre-training tests, the cut-off spatial frequencies^16^,^17^,^18^,^29^,^30^,^31^ under the two conditions were collected, and training sessions with AO correction were performed on consecutive days. A contrast detection task was used for the CSF measurement and perceptual learning task, but at different spatial frequencies^16^,^17^,^18^,^29^,^30^,^31^. In the CSF measurement, seven spatial frequencies (i.e., 0.6, 1, 2, 4, 8, 16, 24 cpd) were included, whereas in the perceptual learning task, the trained spatial frequency was set at the cut-off spatial frequency in the pre-training CSF test with AO correction^18^,^30^.

### Effects of AO correction

**Figure 1:**
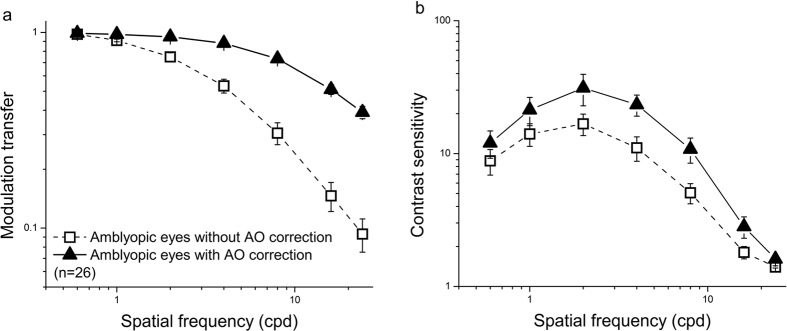
Effects of AO correction on the MTFs and CSFs of amblyopic eyes. MTFs (**a**) and CSFs (**b**) of the amblyopic eyes (n = 26) measured without (dashed curves, open squares) and with (solid curves, solid triangles) AO correction. The error bars indicate the standard errors across observers.

Figure 1a illustrates the average MTFs of the amblyopic eyes at the seven tested spatial frequencies, which were calculated from the wave-front aberrations that we recorded during the contrast sensitivity measurements in the pre-training testing stage. AO correction dramatically improved the MTFs (F(1, 25) = 106.34, partial eta squared = 0.81, p < 0.001). The ratio of the MTF with AO correction to that without AO correction (i.e., when only the sphere and cylinder were corrected by the trial lenses) increased steadily with spatial frequency from 1.022 ± 0.002 at 0.6 cpd to 7.793 ± 1.980 at 24 cpd.

Figure 1b illustrates the average CSFs of the amblyopic eyes as measured with and without AO correction in the pre-training testing stage. An ANOVA test revealed that the CSF with AO correction was significantly greater than that without AO correction (F (1.25) = 10.60, partial eta squared = 0.30, p = 0.003). The gain in contrast sensitivity due to AO correction was defined as the ratio of the contrast sensitivity with AO correction to that without AO correction and increased with spatial frequency up to 4 cpd (from 1.27 ± 0.09 at 0.6 cpd to 2.50 ± 0.35 at 4 cpd) and subsequently decreased with spatial frequency (from 1.78 ± 0.17 at 8 cpd to 1.13 ± 0.06 at 24 cpd). AO correction also significantly increased the cut-off spatial frequency from 10.15 ± 0.95 cpd (mean ± SE) without AO correction to 13.67 ± 0.97 cpd with AO correction (t(25) = 4.75, p < 0.001).

### Improvement at the trained spatial frequency after perceptual learning

**Figure 2:**
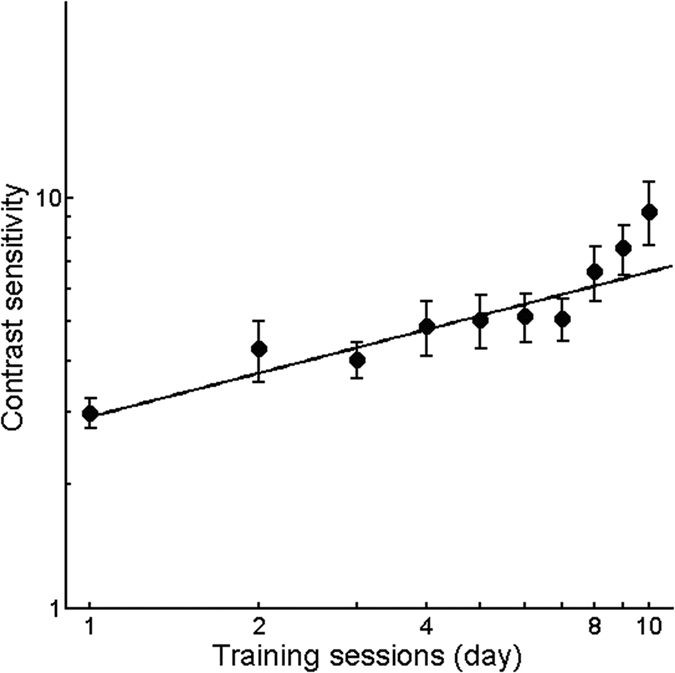
Improvement at the trained spatial frequency after perceptual learning. The average learning curve for the amblyopic eyes (n = 26) for the first 10 training sessions. The black line represents the best linear fit. The error bars indicate the standard errors across observers.

For the trained spatial frequency, training significantly increased the contrast sensitivities of the amblyopic eyes with AO correction (t(25) = 4.47, p < 0.001) by 8.52 ± 1.14 dB or 234% ± 50% (mean ± SE, Equation 1) from 2.50 to 8.34 ± 1.26 (Equation 2). The average learning curve (i.e., contrast sensitivity as a function of the training session) for the first 10 training sessions is illustrated in Figure 2. The slope of the improvement was 0.35 log units per log unit training session (r^2^ = 0.87, p < 0.01).

This training effect was sustained in the natural viewing condition in which the contrast sensitivity at the cut-off spatial frequency without AO correction increased by 9.28 ± 0.79 dB (Equation 1) from 2.50 to 8.04 ± 0.70 or 221% ± 28% (Equation 2).

### Improvement in the CSF after perceptual learning

**Figure 3:**
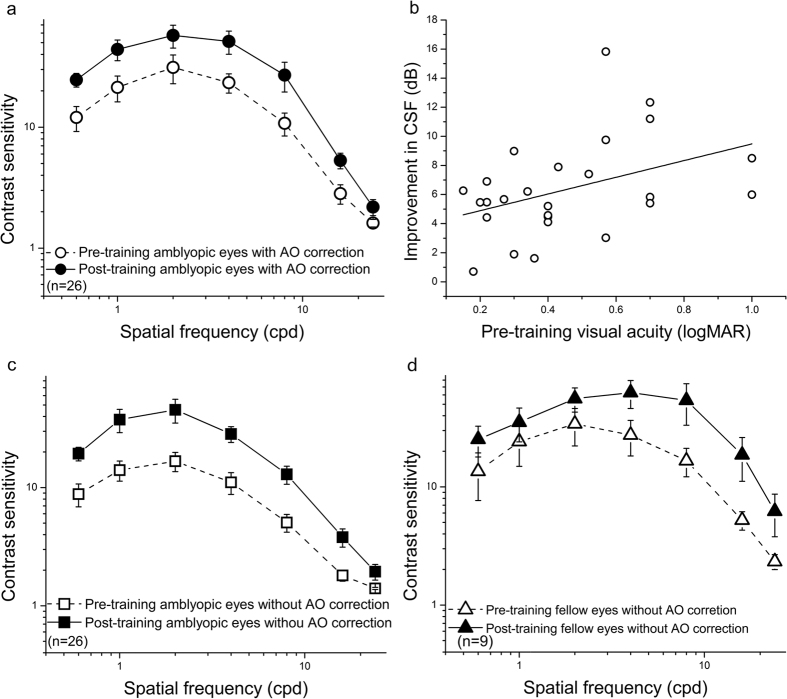
Improvement in the CSF after perceptual learning. (**a**) Pre- (dashed curve and open circles) and post-training (solid curve and circles) CSFs for amblyopic eyes with AO correction (n = 26). (**b**) Average improvements in CSF with AO correction (dB) as a function of pre-training amblyopic VA (logMAR) (n = 26, circles). (**c**) The learning effect with AO correction is maintained under the condition without AO correction for the amblyopic eyes (n = 26, pre-training CSF: dashed curve and open squares, post-training CSF: solid curve and squares). (**d**) The learning effect with AO correction is transferred to the untrained fellow eyes (n = 9, pre-training CSF: dashed curve and open triangles, post-training CSF: solid curve and triangles). The error bars indicate the standard errors across observers.

The average pre- and post-training CSFs with AO correction in the amblyopic eye are illustrated in Fig. 3a. Significant improvements in CSF were observed in the condition with AO correction (F(1, 25) = 23.50, partial eta squared = 0.49, p < 0.001). The average improvements across observers and spatial frequencies were 6.35 ± 0.66 dB (Equation 1) or 197% ± 38% (mean ± SE, Equation 2). The average improvements in CSF were positively correlated with the pre-training amblyopic eye VA (Fig. 3b, r = 0.40, p = 0.04), which suggested greater CSF improvements for those who had initially worse visual acuities. The average improvements in CSF were not significantly correlated with age (r = −0.01, p = 0.973), which suggested that the improvements in CSF induced by perceptual learning are independent of trainee age^33^.

Because visual perception without AO correction (i.e., with spectacle correction only) is more critical in terms of treating amblyopia, we also assessed the training effect on the CSFs under conditions without AO correction. Meanwhile, to assess the interocular transfer of the learning effect, the CSFs of the untrained fellow eyes were also measured without AO correction in 9 patients. We found that the CSFs in the condition without AO correction were significantly improved in both eyes (Fig. 3c, amblyopic eyes: (F(1, 25) = 20.25, partial eta squared = 0.45, p < 0.001; Fig. 3d, fellow eyes: F(1, 8) = 10.10, partial eta squared = 0.56, p = 0.013). The average improvements across observers and spatial frequencies were 7.01 ± 0.82 dB or 159% ± 27% in the amblyopic eyes and 5.26 ± 1.03 dB (Equation 1) or 93% ± 30% (Equation 2) in the fellow eyes. These improvements in the amblyopic eyes under the two conditions were not significantly different (t(1, 25) = 1.19, p = 0.246). These results indicated that the learning effect of perceptual learning was maintained in a natural viewing condition (i.e., without AO correction), and the training benefit in the amblyopic eye did not interfere with the visual perception of the untrained eye.

### Improvement in VA without AO correction after perceptual learning

**Figure 4:**
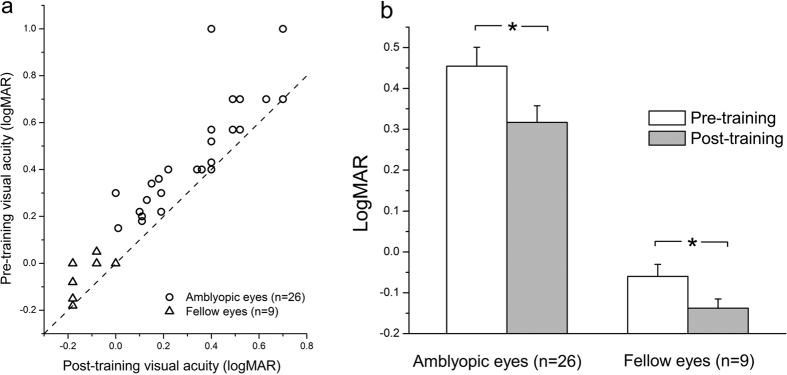
Improvements in VA without AO correction after perceptual learning. (**a**) The pre- and post-training VAs of the amblyopic eyes (open circle, n = 26) and untrained eyes (open triangle, n = 9). The pre-training VA is plotted as a function of the post-training VA. The dashed line indicates the prediction of no improvement. (**b**) The average VAs measured without AO correction before (white bars) and after (grey bars) training in the amblyopic eyes and untrained fellow eyes. The error bars indicate the standard errors across observers; ‘*’ indicates p < 0.05 (2-tailed paired t-test).

The VAs were also improved to differing extents in the majority of our observers (Fig. 4a). The VAs of the amblyopic eyes were significantly improved (t(25) = 3.21, p = 0.004) from 0.45 ± 0.05 (logMAR, mean ± SE) to 0.32 ± 0.04 (Fig. 4b, left) with an average improvement of 2.76 ± 0.48 dB (Equation 1) or 25% ± 3% (Equation 2). The improvements in the VAs of the amblyopic eyes were positively correlated with the pre-training VAs (r = 0.48, p = 0.012), and not with the observer’s age (r = −0.15, p = 0.454). This result was consistent with that found regarding improvements in CSFs, and suggested that greater VA improvements for those who had initially worse visual acuities^15^,^16^ and independent on age^15^. For the 9 observers in whom we conducted additional measures on the untrained fellow eyes, the VAs of the fellow eyes were also significantly improved (t(8) = 3.03 p = 0.016) from −0.06 ± 0.03 (logMAR, mean ± SE) to −0.14 ± 0.02 (Fig. 4b, right) with an average improvement of 1.50 ± 0.49 dB (Equation 1) or 15% ± 5% (Equation 2).

## Discussion

We demonstrated that AO correction successfully improved optical quality by increasing the MTFs of older children and adults with anisometropic amblyopia. AO correction also improved the observers’ CSFs. Training with AO correction using a contrast detection task conducted at the individuals’ cut-off spatial frequencies significantly improved the amblyopic eyes’ contrast sensitivities. This learning effect was maintained in a natural viewing condition involving only spectacle correction (i.e., without the AO correction). Training also improved the amblyopic eyes’ VAs without impairing the untrained fellow eyes’ visual performance including contrast detection or VA.

The improvement in the MTF with AO correction in our study was similar to that previously reported by de Gracia *et al*.^34^ for a 5-mm pupil in normal adults, whereas the improvement in CSF observed here was different. In their report, the increase in contrast sensitivity occurred only at 7.6 cpd or higher, and the greatest improvement was 1.52-fold at 22.7 cpd. These previous results suggest that high spatial frequencies benefit more from AO correction in normal adults^35^,^36^. In our study, AO correction improved the patients’ contrast sensitivities at all of the tested spatial frequencies, and the greatest improvement occurred at 4 cpd. Clearly, the amblyopic eyes could not gain substantial visual benefits from the AO correction at high spatial frequencies. These results are consistent with the high-frequency contrast sensitivity deficits that have previously been found in anisometropic amblyopia using laser-created interference fringes that are largely unaffected by ocular optics, which suggests the reduced contrast sensitivities is due to a neural origin rather than an optical defect^37^.

Training with AO correction dramatically improved the amblyopic eyes’ contrast sensitivities with average improvements of 8.52 ± 1.14 dB at the trained frequency and 6.35 ± 0.66 dB across all of the tested frequencies (0.6 cpd to 24 cpd). These visual benefits were well sustained in the natural viewing condition (i.e., without AO correction); the average improvements in this condition were of 9.28 ± 0.79 dB at the trained frequency and 7.01 ± 0.82 dB across all of the tested frequencies. The training effect also transferred to the untrained VA and the untrained fellow eyes. The patient VAs without AO correction improved by 25%, from 0.45 to 0.32 (logMAR). In the fellow eyes, the contrast sensitivities were improved by 5.26  ± 1.03 dB across observers and spatial frequencies, and the VAs were improved by 15%, from −0.06 to −0.14. These improvements were different from those reported by Rossi and Roorda^38^, who used small letters with a 0.125-arcmin gap as the visual stimuli to train four normal observers with AO correction and found that the pre- and post-training measurements of the visual resolution threshold without AO correction were not significantly different. The possible explanation for these differences was the different training protocol used as a perceptual learning task^8^. Letter identification tasks have been demonstrated to exhibit little or no substantial improvements^39^ and little or no transfer^40^,^41^ with training, whereas a contrast detection task at a given spatial frequency has been demonstrated to exhibit substantial improvement and transfer^16^,^17^,^18^,^29^,^30^,^31^.

Using a training protocol similar to ours that involved a different optical quality during training (i.e., a natural viewing condition), Zhou *et al*.^16^ trained adult amblyopes and observed average improvements of 9.8 dB at the trained frequency and 5.7 dB across all of the tested frequencies. Huang *et al*.^17^ reported averaged improvements of 10.7 dB at the trained frequency and 6.98 dB across all of the tested frequencies. Hou *et al*.^29^ observed average improvements of 9.2 dB at the trained frequency and 6.6 dB across all of the tested frequencies, and Chen *et al*.^31^ observed average improvements of 9.7 dB at the trained frequency. Thus, to some extent, contrast detection training in amblyopia appears to be insusceptible to AO correction. Indeed, in normal adults^18^ and subjects with highly aberrated keratoconic eyes^19^,^20^, perceptual learning with AO correction elicits greater visual neural plasticity than learning without AO correction as indicated by greater contrast sensitivity and VA improvements. One possible explanation for this inconsistency is that the limits of the visual system might vary in different populations. In normal adults and adults with highly aberrated keratoconic eyes, visual processing is more limited by optical factors (i.e., aberrations^42^,^43^), whereas for amblyopes, visual processing is primarily limited by neuronal factors (i.e., factors with neuronal origins^37^). Therefore, AO correction may improve the learning potentials of normal adults and adults with highly aberrated keratoconic eyes without benefiting training in amblyopia. The other possible explanation is the different amblyopic sample characteristics between the present and previous studies, such as the initial VA and sample size. The initial deficit (i.e., initial VA) was positively correlated with the improvement in VA^15^. The average magnitude of the initial pre-training corrected VA of our observers was better than that in previous studies. The sample set in this study contained many more observers than in previous studies.

Our results suggest that AO correction may improve the optical qualities of amblyopic eyes. The effects of perceptual learning with AO correction in amblypoes are maintained in this condition not only with AO correction, but also without AO correction and are also transferred to the untrained VAs and the untrained eyes.

## Methods

### Participants

Twenty-six older children and adults (seventeen females; mean age: 16.2 ± 5.0 years, mean ± SD) with anisometropic amblyopia participated in this study. The participants’ refractive errors, VAs with glasses and treatment histories are listed in Supplementary Table S1. All observers were instructed to wear spectacles, which were prescribed by the first author of this paper, in their daily lives for a period of at least 4 weeks before the measurements.

This research was approved by the ethics committee of West China Medical School, Sichuan University (Chengdu, China). All research activities adhered to the tenets of the Declaration of Helsinki. Informed written consent was obtained from each observer or their legal guardian before the initiation of the experiment.

### Apparatus

A customized AOPL system^28^ was used to measure and correct the HOAs in this study. The AOPL system consisted of a Hartmann-Shack (HS) wave-front sensor with a 16 × 16 microlens array, a charge-coupled device (CCD) camera, a 35-element bimorph deformable mirror, a control system integrated with a PC computer and a visual stimuli-generating system. The HS wave-front sensor was placed adjacent to the observer’s pupil and the deformable mirror and was used to measure the ocular wave-front aberrations. The deformable mirror was used to correct the optical aberrations. The PC-integrated control system was used to control the AO system in a closed-loop fashion. The visual stimuli-generating device was inserted in front of the HS wave-front sensor via a beam splitter, which consisted of a video converter^44^ and an OLED minidisplay. The visual stimuli were generated by another PC running MATLAB 6.5 with the Psychtoolbox^45^,^46^ extensions. The field of view was 1.5° in diameter though a 4-mm artificial pupil. The experiments were performed in a dark room in which the observers’ pupils were naturally dilated without the use any mydriatic. During the experiment, trial lenses were added to correct any defocus and astigmatism based on the individuals’ refraction errors.

### Experimental design

Each participant went through the following four consecutive stages: a pre-training practice stage, a pre-training test stage, a training stage, and a post-training test stage. The first stage was arranged on the first day of the experiment. A 30- to 40-minute practice session was provided to enable the observers to familiarize themselves with the optical system, the task and the keyboard. The practice session included 280 trials, half of which were undertaken with AO correction, and the other half of which were performed without AO correction. These practice trials included all seven spatial frequencies, i.e., 0.6, 1, 2, 4, 8, 16 and 24 cpd, that were used for the CSF measurements; thus, 40 trials were practiced at each spatial frequency. The same task that was used in the testing was used for the practice session, except that the contrasts of the visual stimuli were relatively high (supra-threshold). Normally, the observers finished the practice session with performance levels of 90% or above. For participants with less than 90% accuracy, one additional block was practiced until the accuracy reached or exceeded 90%.

The pre- and post-training test stages included the measurement of the amblyopic eye’s VA and CSF. VA corresponded to 75% correct judgements and was assessed under natural viewing conditions using the Chinese Tumbling E Chart at a distance of 5 m. The VA was converted to units of the logarithm of the minimum angle of resolution (LogMAR). The CSFs of the amblyopic eyes were measured both without and with AO correction via the AOPL system. The CSF measurements in the two AO conditions were conducted on two consecutive days according to a pseudorandom order for each participant, but the pre- and post-training measurement order was fixed for each participant. Additional CSF measurements of the fellow eyes were also conducted in 9 of the 26 participants (subjects 1 to 9); their HOAs were uncorrected to evaluate whether any interocular transfer of the learning effect occurred.

During the training stage, the training sessions were performed on consecutive days in single 40- to 60-minute sessions per day for each participant.

### Contrast sensitivity function (CSF) measurement

Contrast sensitivity was measured at seven spatial frequencies, i.e., 0.6, 1, 2, 4, 8, 16 and 24 cpd, using vertical sine-wave gratings as the visual stimuli. A 0.5-degree half-Gaussian envelope surrounding the grating was added to minimize edge effects. A two-interval forced-choice (2IFC) contrast detection task with a three-down one-up staircase procedure^18^ was used to measure the contrast thresholds, and the reciprocals of the contrast thresholds were defined as the contrast sensitivities. The three-down one-up staircase procedure^47^ involved a decrease in the signal contrast by 10% after every three consecutive correct responses and an increase in the signal contrast by 10% after each incorrect response and converged to a correct performance level of 79.3%.

One CSF testing session consisted of 8 blocks with 77 trials in each block. All of the tested spatial frequencies were even and randomly assigned in different trials in one session; thus, 88 trials were used to measure the contrast threshold at each spatial frequency. Each trial began with the presentation of a fixation cross for 267 ms in the centre of the display that was followed by two 117-ms intervals that were signalled by a brief tone at the beginning of each interval. The two intervals were separated by a 500-ms mean luminance blank screen. The signal sine-wave grating appeared in only one of the two intervals, and the observers were asked to indicate which interval contained the signal by pressing a key on the computer keyboard. A brief tone immediately followed each response regardless of its accuracy. The observers were instructed to take a short rest after finishing each block. The measurements for one CSF lasted approximately 40 to 60 minutes.

### Perceptual learning

The same contrast detection task used for the CSF measurement was used to train the amblyopic eye with HOA correction. Only one spatial frequency was used for the training. The trained spatial frequency was set at the cut-off spatial frequency, i.e., the frequency at which the contrast detection threshold of the amblyopic eye was 0.4 in the pre-training CSF test with AO correction^18^,^30^. The cut-off spatial frequency without AO correction was also collected to evaluate the effect of AO correction on the individual CSFs. The starting contrast of the first training trial was set at 0.4 and set at the contrast of the last trial of the previous training session for the subsequent training sessions.

Each training session contained 7 blocks with 90 trials in each block. The observers were allowed to take an optional rest after finishing each block. A typical training session lasted 40 minutes. Nine to seventeen training sessions were offered to our patients. The learning curve was calculated based on the first ten training sessions.

### Modulation transfer function (MTF) calculation

Because a vertical sine-wave grating was used, the horizontal MTFs were calculated from the wave-front aberrations to quantify the optical system with and without AO correction in this experiment^32^. The wave-front aberrations were measured with an HS wave-front sensor and reconstructed with a description that included 35 Zernike polynomials (up to the seventh order excluding tip and tilt) in accordance with the wave-front standards of the Optical Society of America^48^. The calculation of the MTF was based on standard Fourier optics theories using a 4-mm pupil and a wavelength of 550 nm^49^. The MTF factors of the seven spatial frequencies that were tested in the contrast sensitivity measurement are described.

### Data analysis

Two-tailed paired t-tests were used to assess the effects of AO correction on the cut-off spatial frequencies of the amblyopic eyes, the effects of training on the contrast sensitivities at the trained spatial frequency, and the effects on VA. A within-subject repeated-measures analysis of variance (ANOVA) was used to assess the effects of AO correction on the CSFs and the effects of training on the CSFs.

For each observer, the improvements in VA, contrast sensitivity at the trained spatial frequency and the average CSF were calculated as follows:


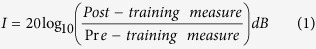


The improvement was also calculated as the percentage change as follows:


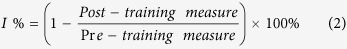


## Additional Information

**How to cite this article**: Liao, M. *et al*. Training to improve contrast sensitivity in amblyopia: correction of high-order aberrations. *Sci. Rep.* **6**, 35702; doi: 10.1038/srep35702 (2016).

## Supplementary Material

Supplementary Information
