# Supplementary material for: Training to improve contrast sensitivity in amblyopia: correction of high-order aberrations
Source: Sci Rep. 2016 Oct 18;6:35702. doi: 10.1038/srep35702 (PMC5067678; doi:10.1038/srep35702)
Supplement: Supplementary Information [file srep35702-s1.pdf]

Supplementary Information:

Training to improve contrast sensitivity in amblyopia: correction of high-order aberrations

Meng Liao, Haoxing Zhao, Longqian Liu, Qian Li, Yun Dai, Yudong Zhang, Yifeng Zhou

Subject	name	Age(years)/Sex	Refraction		Visual acuity (logMAR)		Training spatial frequency(c/deg)	Training sessions
			Amblyopic eye	Fellow eye	Amblyopic eye	Fellow eye		
1	LJN	13/F	+8.00/+0.50×90	+0.50	1	0	8	9
2	YZ	11/F	+4.00/+0.50×85	plano	1	0	10	10
3	ZCY	15/M	+4.00/+3.00×100	-1.00×180	0.57	0	3.5	9
4	PJY	14/M	+2.50/+0.50×165	-2.50	0.43	-0.15	8	10
5	LC	14/F	+4.50/+1.50×75	-0.50/-0.50×180	0.30	-0.18	16	17
6	ZYJ	23/F	+3.00/+1.00×90	-1.75	0.22	0.05	18	10
7	GF	16/F	+2.25/+0.50×90	-1.75/0.50×5	0.3	0	12	9
8	SJY	14/F	+3.50/+1.50×95	-1.00×170	0.22	-0.08	18	9
9	TGW	14/M	+4.50/+2.00×90	-1.00	0.57	-0.18	9	11
10	XQ	12/F	+1.00/+2.00×85	-0.5/-1.00×10	0.40	0.05	15	10
11	LHH	19/M	+4.50/+1.00×90	+2.25/+1.00×70	0.70	0.00	10	9
12	ZWY	14/F	+4.00/+2.00×90	-3.50/0.50×180	0.52	0.00	13	10
13	TJ	26/F	+1.50/+2.00×105	-1.50	0.12	-0.06	22	10
14	LFT	11/M	+3.00/-3.50×180	plano	0.15	-0.08	24	10
15	ZCH	12/M	-3.50/-3.50×10	+0.50	0.18	0.03	16	10
16	ZM	14/F	+5.50	plano	0.70	0.00	13	10
17	LD	15/M	+0.50/+2.50×90	+0.50/+0.50×90	0.57	-0.08	16	10
18	LXC	12/M	+5.00	-0.50	0.36	-0.08	13	10
19	SLL	18/F	+5.00/+0.75×110	-0.50×160	0.70	-0.03	15	10
20	LYY	20/F	+6.00/+1.50×50	+5.00/+2.75×120	0.40	0.10	8	10
21	LYB	26/M	+10.00/+2.00×90	plano	0.40	-0.14	20	11
22	LYQ	13/F	+4.50	plano	0.34	-0.08	12	10
23	WYT	14/F	+3.00/+2.00×110	-0.75	0.40	-0.15	12	10
24	GWL	14/F	+5.00/+2.00×80	-0.50/-0.50×165	0.70	-0.08	8	9
25	TYY	30/F	+5.00/+1.50×180	+0.50	0.27	-0.08	18	13
26	LJH	17/F	+4.00/+1.00×180	+3.00	0.20	0.00	20	9

Table S1. Clinical summary of the subjects.
